# Therapeutic Challenges for Elderly Patients with Primary Hyperparathyroidism

**DOI:** 10.1155/2019/4807081

**Published:** 2019-10-23

**Authors:** Kenneth Sluis, Hyon Kim, Yuling He, Beatrice Wong, Xiangbing Wang

**Affiliations:** Division of Endocrinology, Metabolism, and Nutrition, Department of Medicine, Rutgers University-Robert Wood Johnson Medical School, New Jersey, NJ 08903, USA

## Abstract

Primary hyperparathyroidism (PHPT) predominantly affects older adults, and parathyroidectomy can achieve definitive cure in symptomatic PHPT and asymptomatic meeting surgical criteria. As the population continues to age, the treatment of PHPT in octogenarians and nonagenarians presents a clinical conundrum. This case series presents the management of eight patients 85 years of age and older diagnosed with PHPT. A retrospective chart review of patients diagnosed with primary hyperparathyroidism were identified in a single institution. Those patients 85 years of age and older who were followed up for over one year were included in this case series. The literature on treatment options for this age group was also reviewed. Eight cases of PHPT patients aged 88 ± 2.5 years old with a follow-up average of 5.6 ± 4.4 years were reported in our case series. Six PHPT patients were medically managed and two PHPT patients underwent parathyroid resection. Most of the medically managed PHPT patients except for one had long-term stability of disease for over five years. The treatment of PHPT diagnosed in patients over 85 years of age presents a clinical challenge for which there is no clear consensus guideline. Our case series supports that medical therapy is a feasible option for PHPT patients over 85 years old.

## 1. Introduction

Primary Hyperparathyroidism (PHPT) is a common disease that increases in prevalence with advancing age. Prevalence peaks in women around 70–79 years of age (492 per 100,000) and in men over 80 years old (264 per 100,000) [1]. Most patients with PHPT initially present with asymptomatic hypercalcemia. Those that are symptomatic present with axial and extremity fractures, nephrolithiasis, and psychiatric disturbance [2]. Surgical resection of the involved glands is the standard of care for young or symptomatic patients with PHPT. Medical management is used as a bridge to surgery or in patients who refuse or cannot safely undergo surgery. Strategies include hydration with expectant management, stopping offending agents such as calcium supplementation and medications (thiazides, lithium) and using diuretics, bisphosphonates, selective estrogen receptor modulators, and cinacalcet [2]. Expectant management involves serial monitoring for worsening of symptoms without specific therapeutic intervention [3, 4].

Most of the literature on the treatment of PHPT is not age-specific. About 1.5% of patients over the age of 70 have PHPT [5]. As our population continues to age and as average life expectancy increases, the proportion of elderly patients with PHPT will also grow. Though the standard of care to manage PHPT is surgery to achieve cure, the role of surgery is less clear in the very elderly. The objective of our study is to report eight cases of PHPT patients over 85 years of age who were managed using medical or surgical treatment to elucidate differences in prognosis and outcomes.

## 2. Cases Presentation

We conducted a retrospective chart review of PHPT patients evaluated at Robert Wood Johnson University Hospital from January 2000 to September 2016. Our institution's IRB provided approval for our investigations. There were 556 patients diagnosed with PHPT during this time. The diagnostic criteria of PHPT included: (1) intact PTH > 65 pg/mL (normal range: 10–65 pg/mL) or inappropriately normal levels in the presence of elevated serum calcium, (2) serum calcium > 10.6 mg/dL (normal range: 8.5–10.4 mg/dL), and (3) 24-hour urinary calcium > 100 mg/day (normal range: 100–250 mg/day). Eight PHPT patients aged 85 years and older with follow-up duration longer than one year were included for this study. Patient demographics, baseline characteristics, clinical presentation and overall clinical follow-up were examined.

Eight patients diagnosed with PHPT at the age of 85 years or older were included. The average age at diagnosis was 88 ± 2.5 years old. The average follow-up duration was 5.6 ± 4.4 years. The average calcium was 11.3 ± 0.78 mg/dL, iPTH was 166 ± 154 pg/mL and 24-hour urine calcium was 183 ± 35.5 mg at the time of diagnosis. All eight patients met guidelines [2, 5] for surgery by the serum calcium levels >1 mg/dl upper limit of normal, creatinine clearance <60 cc/min or *T*-score <2.5 (Table 1). Six PHPT patients were medically managed for one to eight years and two PHPT patients underwent parathyroid resection. The surgical group had a pre-operative serum calcium of 12.3 mg/dL while the medically managed group had an average of 10.9 mg/dL. For two patients treated surgically, one patient was cured with normal calcium and PTH levels but was only followed up for two years. One patient had recurrence of PHPT five years after surgery and required medical management with cinacalcet, loop diuretic, and bisphosphonate for ten years. Six patients were managed medically for an average of 5.2 years with minimal complications. Only one of the six patients experienced PHPT-related mortality. This patient had severe dementia, was on medical treatment for eight years, and passed away on hospice after a hip fracture. Of the other medically managed patients, three patients were managed with pharmaceutical therapy, one with bisphosphonate and two with cinacalcet. Two other patients were treated with nonpharmaceutical expectant management, which included oral hydration, discontinuation of HCTZ and maintaining physical activity. They had favorable outcomes after three to six years.

Patient vignettes are demonstrated in Table 1.

## 3. Discussion

The Endocrine Society consensus guidelines recommend parathyroidectomy for both symptomatic and asymptomatic PHPT. Surgery is readily offered for biochemical cure in younger, symptomatic or asymptomatic patients with PHPT meeting surgical criteria [2]. The benefits of parathyroidectomy include advantages in survival, increased bone density, reduced fatigue, and other subjective measures [6, 7].

Much of the current literature advocates for more aggressive surgical therapy for the elderly. However, the benefit of surgery at achieving cure for octogenarians and nonagenarians is less straightforward than for the young. There is increased risk for complications, prolonged operation time and increased length of stay in hospitals for patients over the age of 80 years [7, 8]. As our understanding of PHPT has evolved, different phenotypes of the disease have been recognized, the symptoms of which can be confounded by the aging process in this population [9]. Studies have shown that medically managed patients with PHPT could have stable disease for over ten years [9, 10]. Our expanded case series supports medical management as a reasonable option in the very elderly, especially those with comorbidities.

Literature in favor of medical management of the very old shows promise despite its smaller scope. Khan et al. [4] and Marcocci et al. [11] reviewed the rationale and evidence behind medical management and expectant monitoring. They found that medical management can be effective, and that patients' quality of life can be similar to those treated surgically. Jacobs et al. [3] described four patients with PHPT aged 79 to 87 years treated with medical management as a bridge to surgery or as sole therapy in poor surgical candidates or those refusing surgery. They employed saline hydration, pamidronate and cinacalcet. Medical therapy was a successful bridge to surgical cure with parathyroidectomy for two patients after two weeks to two months of treatment. Medical therapy was not tolerated for another and pursued indefinitely for the final patient. Wong reported two PHPT patients over the age of 90 treated with oral rehydration, bisphosphonate or cinacalcet. They experienced no PHPT related complications or fractures in a follow-up duration of seven months to four years [12].

The limitations of our study include small sample size and short duration of follow-up. One patient had baseline dementia, and 3 nonagenarian patients passed away after 6–10 years follow up, we did not have the data and therefore cannot compare quality of life, cognitive and muscle function before and after intervention.

In summary, our data suggest that medical therapy is a reasonable option for PHPT patients over 85 years old. As life expectancy increases and more patients are diagnosed with PHPT later in life, further consensus guidelines may need to specifically address treatment recommendations for patients over the age of 85 years. Studies with larger patient populations and randomized controlled trials are needed to support our findings.

## Figures and Tables

**Table 1:** Eight cases of PHPT in patients over age 85.

Case #	Clinical description
Case 1	An 86-year-old woman with a history of osteopenia, hyperlipidemia, and GERD was diagnosed with PHPT with a calcium level of 13 mg/dl, iPTH level of 66 pg/ml, 25OHD 34 ng/ml and creatinine 1.07 mg/dl. She underwent surgery and had successful removal of a right upper parathyroid gland. She achieved surgical cure with normalized calcium and iPTH levels. Her last calcium was 9.7 mg/dl, iPTH 12.4 pg/ml and 25OHD 54 ng/ml at her 2 year follow up visit.
Case 2	A 91-year-old woman with a history of hyperparathyroidism with parathyroidectomy of 3.5 glands 5 years ago, hypothyroidism, osteoporosis, and goiter presented with a calcium level of 11.6 and iPTH level of 500. She was diagnosed with relapse of PHPT, and was managed with cinacalcet, furosemide, and risendronate. Serum calcium was controlled for about 8 years, but creatinine started to rise. Ten years after the recurrence of PHPT, cinacalcet had to be lowered and her bisphosphonate had to be discontinued after a further increase in creatinine. She suffered a left hip fracture and a possible aspiration event during the subsequent hospitalization. She passed away from sepsis after 10 years' medical treatment of recurrence of PHPT.
Case 3	An 85-year-old woman with a history of a resected follicular Hurthle cell neoplasm presented with a calcium level of 11.5 mg/dl and iPTH of 279 pg/ml. She was diagnosed with PHPT and treated with cessation of hydrochlorothiazide, initiation of furosemide, and cinacalcet for 5 years. Cinacalcet dosage had to be increased after a year, but then subsequently discontinued due to worsening renal function (Cr 2.98–3.3 mg/dl). Five years after diagnosis her PTH level increased (483–511 pg/ml) but calcium was 10–11 mg/dL. She was hospitalized for cellulitis 6 years later and was discharged to hospice care for end-stage renal disease. She passed away at the age of 91.
Case 4	A 91-year-old man with a history of type 2 diabetes, hyperlipidemia, dementia, atrial fibrillation, and congestive heart failure was hospitalized for sepsis. He was found to have a calcium level of 11.1 mg/dl, iPTH 59 pg/ml, 24-hour calcium 203 mg, and 25OHD 26 ng/ml. He was diagnosed with PHPT. He was treated with calcitonin, oral hydration and furosemide. He was discharged to his long-term care facility. His calcium level remained normal up to 10.9 mg/dL with Cr 1.8 mg/dl at 1-year follow up.
Case 5	A 96-year-old woman with a history vitamin D deficiency, osteoporosis, and hypothyroidism was found to have a calcium level of 11.2 mg/dl, iPTH 165 pg/ml, and 24-hour urine calcium of 204 mg. A parathyroid scan showed a possible left lower pole adenoma. She refused surgery and was treated with zoledronic acid infusion for her osteoporosis. Calcium decreased to 10.2 mg/dL three years after diagnosis she was given a second zoledronic acid infusion. She was followed for 4 years and did well with serum calcium maintained from 10.2 to 10.7 md/dL and iPTH 147 pg/ml.
Case 6	An 86-year-old woman with a history of osteoporosis, hypertension and dementia was found to have a calcium level of 11.1 mg/dl, iPTH 100 pg/ml, and 25OHD 32 ng/mL. She did not tolerate bisphosphonate therapy and was treated with oral hydration, cessation of thiazide diuretic, and encouragement of physically activity. Two years after diagnosis, calcium levels were maintained in the high-normal range (10.2–10.5 mg/dl). Her dementia worsened and she suffered a left hip fracture 8 years after diagnosis. She passed away on hospice from a gangrenous wound after 8 years of expectant medical management of PHPT at age of 96.
Case 7	An 86-year-old woman with osteopenia treated with bisphosphonates and a selective estrogen receptor modulator, hypothyroidism, vitamin D deficiency and DVT was found to have a calcium level of 11.8 mg/dl, iPTH 128 pg/ml, 24-hour urine 142 mg, and 25OHD 31 ng/mL Sestamibi scan did not locate an adenoma. Expectant management with increased oral hydration, decreased milk intake, cessation of thiazides, and encouragement to increase activity was begun. Her calcium rose as high as 12.9 mg/dL but then settled to 11.2 mg/dL on subsequent measurement. She was followed without incident for 3 years.
Case 8	An 89-year-old woman with a history of hypothyroidism was found with a calcium level of 11.5 mg/dl, iPTH 95 pg/ml and 25OHD 24.6 ng/mL. She was asymptomatic and was managed expectantly with oral hydration and increased physical activity. Calcium was maintained from 10.2to 10.9 mg/dL and PTH 77–107 pg/ml. She was hospitalized for hypertensive urgency but otherwise had no sequelae for 6 years.

Note: PHPT = Primary hyperparathyroidism, iPTH = Intact parathyroid hormone, 25OHD = 25 hydroxyvitamin D.
